# First person – Camilla Elinor Korsvig-Nielsen

**DOI:** 10.1242/bio.045120

**Published:** 2019-06-15

## Abstract

First Person is a series of interviews with the first authors of a selection of papers published in Biology Open, helping early-career researchers promote themselves alongside their papers. Camilla Elinor Korsvig-Nielsen is first author on ‘Eyes and negative phototaxis in juvenile crown-of-thorns starfish, *Acanthaster* species complex‘, published in BiO. Camilla Elinor conducted the research described in this article while a bachelor's student in Anders Garm's lab at University of Copenhagen and Mike Hall and Cherie Motti's lab at Australian Institute of Marine Science after a semester at The University of Queensland. She is now a master's student in the lab of Anders Garm at University of Copenhagen, investigating marine invertebrate sensory behaviour and ecology with emphasis on vision and olfaction.


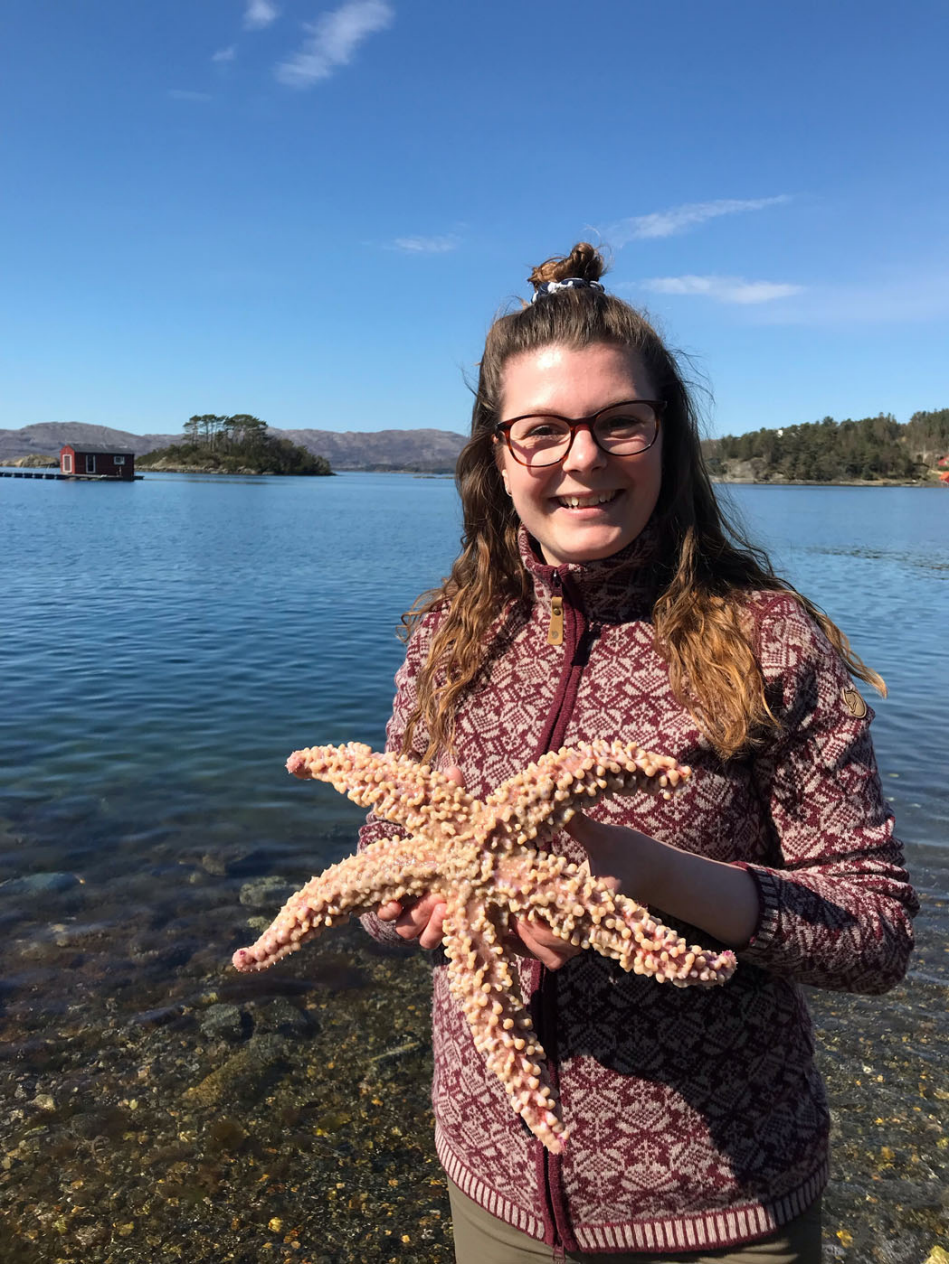


**Camilla Elinor Korsvig-Nielsen**

**What is your scientific background and the general focus of your lab?**

I have a bachelor's degree in biology and I am currently doing my master's thesis about starfish sensory behaviour, as a collaboration between the University of Copenhagen and the University of Bergen. I want to investigate the sensory hierarchy of three species of starfish by combining visual and olfactory stimuli and further investigate the olfactory nerves and eyes. This paper is a result of my bachelor's thesis, which I carried out in Australia after a semester at The University of Queensland. I quickly realized during my stay at Australian Institute of Marine Science that I wanted to continue within research. During the first year of my master's, instead of taking a course, I did a small research project in Bergen, Norway, about the deep-sea shark parasite, *Anelasma squalicola*. Here, the focus was settling patterns and morphology. I am primarily interested in marine invertebrates, and in my research I mainly focus on sensory behaviour and structures (eyes and ‘noses’), though also on more general morphology and neurobiology.

**How would you explain the main findings of your paper to non-scientific family and friends?**

Crown-of-thorns starfish (COTS) use vision to locate their home and prey – the coral reef. Our findings on these young starfish show that the coral reef needs to be 40 cm or bigger before the starfish can see it and move against it. When presented with anything smaller, the starfish walked around randomly and could not find ‘home’. This is one of the simplest visual tests, as they were just given the choice between coral reef and open water. We also tested for spatial vision, which means being able to separate two objects from each other. Here we only saw indications of this kind of vision. As earlier studies have shown the adult starfish being able to differentiate two objects, we deduced that COTS develop their eyes, and hence vision, with age. These results indicate that the young starfish probably use their vision as a way of ensuring they stay on/inside the coral boulder.

**What are the potential implications of these results for your field of research?**

These findings are important for management of these coral-eating starfish. They are a pest to coral reefs around the tropics when occurring in large densities; knowing how the juveniles perceive the reefs hopefully makes it easier to find a method to successfully remove them from the reef. The juveniles are quite elusive and normally hide within the coral boulders, so when divers are on a removal mission, it is very difficult to get the juveniles. By targeting the juveniles before they reach sexual maturity, the amount of COTS will automatically decrease with time.

**What has surprised you the most while conducting your research?**

I am relatively new into the whole research field, with this study being my first, and I think I was surprised and astonished by almost everything. When I was little, I was always curious about everything alive, so during this research I was particularly surprised by how you can study animal behaviour. It took so much time and creative thinking together with a lot of handy skills, which is one of the things I love most about science – being able to combine different skills.

**Figure:**
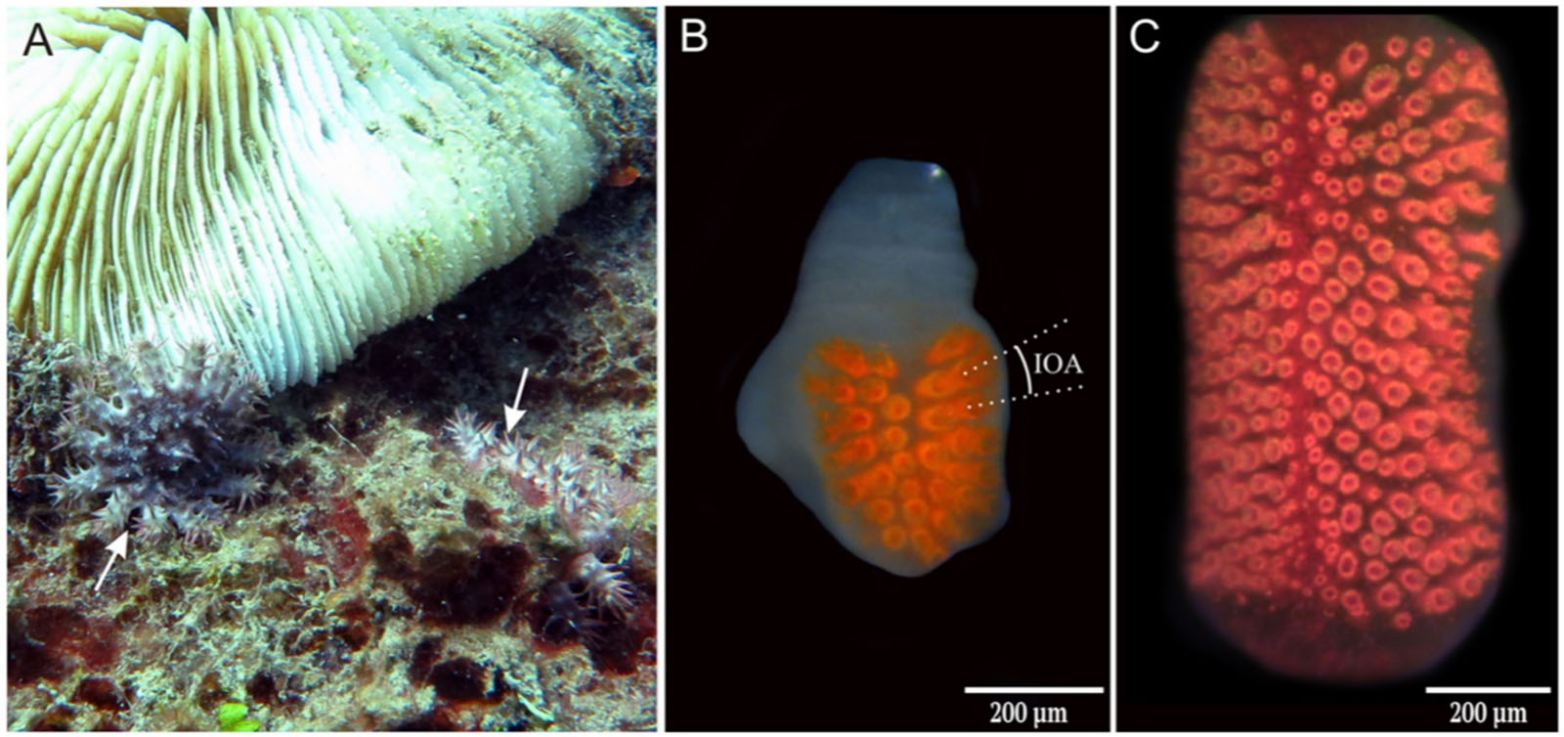
**Compound eyes of COTS.** (A) Two juvenile COTS (white arrows) sitting close to a Fungia coral. (B) Compound eye from a juvenile showing the interommatidal angle (IOA). (C) The compound eye of an adult COTS.

“It took so much time and creative thinking together with a lot of handy skills, which is one of the things I love most about science.”

**What changes do you think could improve the professional lives of early-career scientists?**

Personally, I find it very stressful and almost impossible to find a PhD position. I mean, where to look? There are many opportunities in different companies, universities and so on. It is difficult to just do a broad Google search. Also, many of the posted positions are already designed for another person, though you do not know that when applying, so you just have to hope. Sadly, I think many people choose another career, as it can seem too difficult to figure out what it means to do a PhD, how and where to apply. It would make it so much simpler for early-career scientists to have more guidance and a better overview of where to look for PhD positions. Maybe even better would be the option to easily get funding to design your own PhD together with a supervisor of your choice.

**What's next for you?**

I am still finishing my master's, which will be complete in February 2020, after that I want to continue my career within the research field. I hope and have my fingers crossed that I'll get into a PhD within marine biology. As it can be quite difficult to find the right PhD position, I am currently also applying for jobs within the field, though my top priority is a PhD.
